# Pharmacokinetics of lotilaner following a single oral or intravenous administration in cats

**DOI:** 10.1186/s13071-018-2966-6

**Published:** 2018-07-13

**Authors:** Céline E. Toutain, Wolfgang Seewald, Martin Jung

**Affiliations:** Elanco Animal Health, Mattenstrasse 24a, CH-4058 Basel, Switzerland

**Keywords:** Lotilaner, Isoxazoline, Pharmacokinetics, Cat, Oral, Intravenous, Food effect, Fed, Fasted

## Abstract

**Background:**

Credelio^TM^ (lotilaner) is an oral ectoparasiticide from the isoxazoline class developed for the treatment of flea and tick infestations in cats. It is formulated as a pure *S*-enantiomer in flavoured chewable tablets. The pharmacokinetics of lotilaner were investigated after intravenous or oral administration and under fed or fasted conditions in cats. Twenty-six adult cats were enrolled in a pharmacokinetic study evaluating either intravenous or oral administration of lotilaner. Following the oral administration at a dosage of 6 mg/kg, under fed or fasted conditions, or intravenous administration of 3 mg/kg, blood samples were collected up to 35 days after treatment. Lotilaner blood concentrations were measured using a validated liquid chromatography/tandem mass spectrometry method. Pharmacokinetic parameters were calculated by non-compartmental analysis. In addition, *in vivo* enantiomer stability of lotilaner was evaluated in a separate bioanalytical study.

**Results:**

Following oral administration in fed cats, lotilaner was readily absorbed and peak blood concentrations reached within four hours. The terminal half-life was 33.6 days. Food enhanced the absorption, providing close to 100% oral bioavailability and reduced the inter-individual variability. Following intravenous administration, lotilaner had a low clearance of 0.13 l/kg/day, large volumes of distribution V_z_ and V_ss_ of 5.34 and 5.37 l/kg, respectively and a terminal half-life of 28.7 days. In addition, there was no *in vivo* racemization of lotilaner.

**Conclusions:**

The pharmacokinetic properties of lotilaner administered orally as a flavoured chewable tablet (Credelio^TM^) were studied in detail. With a T_max_ of 4 h and a terminal half-life of 33.6 days under fed conditions, lotilaner provides a rapid onset of flea and tick killing activity with consistent and sustained efficacy for at least one month in cats.

**Electronic supplementary material:**

The online version of this article (10.1186/s13071-018-2966-6) contains supplementary material, which is available to authorized users.

## Background

The new ectoparasiticide lotilaner, a member of the isoxazoline class, was developed for dogs as a pure *S*-enantiomer in flavoured chewable tablet (Credelio^TM^) [1]. Credelio^TM^ is highly efficacious against fleas and a wide variety of ticks on dogs [2–9] and has now been developed for cats. At the time this manuscript was written, no oral isoxazoline product was commercially available for use in cats, while several were available for dogs [10–12]. Lotilaner is an antagonist of γ-aminobutyric acid (GABA)-gated chloride channels [13]. It has a molecular weight of 596.76, a measured log Pow (octanol/water partition coefficient) of 5.3 and it is highly bound to plasma proteins. The pharmacokinetics of lotilaner were determined in dogs after intravenous and oral administration and under different feeding regimens [14]. Following oral administration to fed dogs, lotilaner displayed a terminal half-life (T_1/2z_) of 30.7 days and maximum blood concentrations were reached within two hours. Food enhanced the absorption, providing an oral bioavailability above 80% and reduced the inter-individual variability. In addition, the time of feeding with respect to dosing (within 30 min of dosing) or the reduction of the food ration to one-third of the normal daily ration did not impact bioavailability. Following intravenous administration, the T_1/2z_ was 24.6 days, the clearance was low (0.18 l/kg/day) and the volume of distribution was large (6 l/kg) [14]. Lotilaner was subsequently developed in a unique formulation for cats, as an oral flavoured chewable tablet. The flavoring (yeast and vanillin) was specifically chosen for cats and the tablets are of small size and available in two different strengths (12 and 48 mg; 5.0 and 7.5 mm, respectively), to deliver a dose of 6–24 mg/kg. Laboratory and field studies demonstrated that it provides fast and sustained efficacy against fleas (*Ctenocephalides felis* and *Ctenocephalides canis)* and ticks (*Ixodes ricinus*) [15–17]. Repeated elevated doses administered orally monthly to young cats at 8 weeks of age demonstrated that, at the maximum dose rate of 24 mg/kg/month, lotilaner has a wide margin of safety [18]. The lower therapeutic dose in cats compared to dogs (minimum recommended dose rate of 6 *vs* 20 mg/kg) is not related to safety concerns, but is efficacy-driven. *Ixodes ricinus*, the most important tick species in Europe, was found to be highly susceptible to lotilaner on cats, even at the dose rate of 6 mg/kg. Hence, this dose rate enabled the development of attractive small-sized tablets for cats providing one-month full efficacy against fleas as well as *Ixodes ricinus* ticks.

Credelio^TM^ provides one-month effectiveness with a fast onset of action killing fleas and ticks [16]. This manuscript presents the study performed in cats to determine the pharmacokinetic profile after intravenous and oral administration, and to describe the effect of feeding on oral pharmacokinetics in cats.

## Methods

### Animal management

European shorthair adult cats weighing 2.60 to 5.60 kg (mean ± standard deviation (SD): 3.86 ± 0.94 kg) and aged 9 to 21 months (mean ± SD: 11 ± 4 months) were included. Each animal was uniquely identified and acclimatized to the study conditions for at least 2 weeks. Only healthy animals were included and suitability was evaluated by physical examination and clinical pathology. Cats were housed indoors, in climate-controlled facilities in accordance with accepted Swiss cantonal laboratory animal care and use guidelines. They were kept in small groups except for the day of treatment administration where cats were housed individually, to avoid potential cross-contamination between animals and to monitor for potential adverse events. They were fed once daily with an appropriate ration of a dry commercial feline feed and water was available *ad libitum*. Cats were observed for general health, behaviour and appetite at least once daily throughout the study. All animals returned to their normal housing facilities on completion of the study.

### Experimental design

The objective was to determine the pharmacokinetics of lotilaner after a single intravenous or oral administration, under fed or fasted conditions. Twenty-six adult cats (13 males and 13 females) were allocated to three treatment groups, each containing males (M) and females (F) as follows: one intravenous group of eight cats (4M/4F) treated under fasted conditions; one oral group of twelve cats (6M/6F) treated 30 ± 5 min after feeding (dry food); and one oral group of six cats (3M/3F) treated under fasted conditions. In the unfed groups (intravenous and oral-fasted), feeding took place approximately 4 h after treatment. Each cat from the oral groups received a single administration of the final tablet formulation at the target minimum dose of 6 mg/kg lotilaner, which was the final minimum therapeutic dose. Each cat from the intravenous group received a single administration of lotilaner in a solution consisting of 23% w/v physiological saline and tetraglycol ad 100% w/v, at the target dose of 3 mg/kg lotilaner, which was a dose known to be well tolerated intravenously. Blood specimens were collected from the jugular vein in K3-EDTA tubes at pre-dose and at 5 min (intravenous only), 30 min, at 1, 2, 4, 8, 24, 48 and 72 h and 7, 14, 21, 28 and 35 days post-treatment. Whole blood specimens were stored frozen at approximately -20 °C until analysis with a validated liquid chromatography/tandem mass spectrometry (LC-MS/MS) method.

### Analysis of lotilaner in blood

Lotilaner was quantitatively analyzed in whole blood using the same analytical method involving LC-MS/MS as previously used in dogs [14]. Whole cat blood samples (80 μl) were extracted by precipitation with acetonitrile and further diluted with acetonitrile. A proprietary closely related chemical analogue was used as the internal standard. Ten microliters of each diluted supernatant were chromatographed by high-performance liquid chromatography (HPLC) on a reversed-phase column [Thermo Betasil C18, 5 μm (50 × 4.6 mm)] with an isocratic mobile phase consisting of 0.1% formic acid and acetonitrile (15:85 v:v) using a flow rate of 0.8 ml/min and quantitatively analyzed on an API 5000™ triple quadrupole mass spectrometer system (AB Sciex Germany GmbH, Darmstadt, Germany) using the negative Turbo IonSpray ionization mode and multiple reaction monitoring of the transition m/z 596 to 181 for lotilaner.

The method was validated over a linear range of 7 to 7000 ng/ml, with a lower limit of quantification (LLOQ) of 7 ng/ml, according to Food and Drug Administration (FDA) and European Medicine Agency (EMA) guidelines [19, 20]. Mean inter-day precision was 9.3% at LLOQ and ranged between 3.0–10.4% at the other levels and the mean inter-day accuracy ranged between 99.7–107.2%. In addition, specificity, dilution integrity, recovery and matrix effect, carryover, and stability in matrix and solutions were established. Long-term stability in frozen blood at -20 °C was demonstrated over 9 months.

### Enantiomeric stability

The *in vivo* enantiomeric stability of lotilaner was investigated in a separate bioanalytical study. Blood specimens from 16 adult cats which had received a single oral administration of the pure enantiomer drug at 5 mg/kg (tablets, during a pilot efficacy study) were analyzed at four time points (4 h and 28, 56 and 91 days post-dosing) using an enantioselective analytical method. This method involved precipitation of 200 μl whole blood with acetonitrile and subsequent solid phase extraction on C18 cartridges, evaporation to dryness and reconstitution in heptane/ethanol 4:6, v/v. Enantiospecific analysis was carried out by chiral normal phase HPLC using a Daicel Chiralpak IA-3 column (150 × 4.6 mm) and a mobile phase consisting primarily of heptane and isopropanol. Mass spectrometric detection was performed on an API 4000 Qtrap triple quadrupole instrument (AB Sciex) using the negative Turbo IonSpray ionisation mode and multiple reaction monitoring.

### Pharmacokinetic and statistical analysis

Pharmacokinetic parameters were calculated for individual animals using non-compartmental analysis. The validated statistical software SAS®, version 9.2.2, was used for all calculations. The peak blood concentration (C_max_) and time to peak concentration (T_max_) were observed values, for the oral groups. The terminal half-life (T_1/2z_) was calculated by log-linear regression over a suitable time interval. The area under the concentration curve (AUC) between 0 and the last time point where the blood concentration was above the limit of quantitation (AUC_last_), was calculated by the linear trapezoidal rule and values below the limit of quantitation at the beginning of the profile were treated as zero. The area under the concentration curve from zero to infinity (AUC_inf_) was the sum of AUC_last_ and the extrapolation after the last observed timepoint; the second term was calculated by log-linear extrapolation from the last observed time point to infinity, using the T_1/2z_. The mean residence time (MRT) was calculated as the ratio of AUMC/AUC, where AUMC is the area under the first moment curve.

The clearance per kilogram of body weight (CL), defined as dose per kilogram of body weight/AUC, the volume of distribution at steady-state per kilogram of body weight (V_ss_), which is CL × MRT and the apparent volume of distribution per kilogram of body weight (V_z_), which is CL × T_1/2z_ / ln(2), were determined for the intravenous group only.

Bioavailability (F%) in the oral groups was determined as (geometric mean of dose-normalized AUC_last_ in the oral group) / (geometric mean of dose-normalized AUC_last_ in the intravenous group). In this study, AUC_last_ was also equal to AUC from 0 to 35 days (AUC_0-35 d_). AUC_inf_ was found to be an unsuitable parameter for the evaluation of bioavailability because it was not accurate due to the high percentage extrapolated beyond the last measured data point.

A one-way analysis of variance (ANOVA) was performed on log-transformed dose-normalized C_max_ and AUC parameters, with treatment as fixed effect. The mean and the 90% confidence interval (CI) for the difference between two treatment groups was calculated on the log scale and then back-transformed to the original scale, leading to the ratio between the two groups of C_max_ or AUC. The difference (on the log-scale) between two treatment groups can be tested *versus* zero in a t-test (degrees of freedom given in subscripted parentheses after the symbol *t* in the tables, e.g. *t*_(22)_ meaning a *t*-value with 22 degrees of freedom).

French translation of the Abstract is available in Additional file 1.

## Results and discussion

### Enantiomeric stability *in vivo*

No racemization of lotilaner was observed in any of the samples from the 16 cats, demonstrating absence of *in vivo* racemization in cats after administration of pure lotilaner enantiomer (enantiomeric purity ≥ 98.0%). Therefore, as in dogs [14], in the absence of *in vivo* racemization, the investigation of the pharmacokinetics and safety of the opposite (inactive) enantiomer can be waived. Moreover, this justifies the usefulness of lotilaner being administered as single enantiomer, as opposed to the racemate, as well as the use of a non-enantioselective analytical method for routine blood analysis for pharmacokinetic purposes.

### Pharmacokinetics of lotilaner and effect of feeding in cats

Whole blood, rather than plasma, has been used to establish the pharmacokinetics of lotilaner in cats and dogs. Due to its excellent long-term storage stability in frozen blood samples (at least 9 months), this enabled not only efficient sample processing (no need to prepare plasma), but also the understanding of the pharmacokinetics in whole blood, which represents the real circulating body fluid and is also the fluid ingested by the blood-sucking parasites, hence is perfectly suited for pharmacokinetic/pharmacodynamic work as well.

**Fig. 1:**
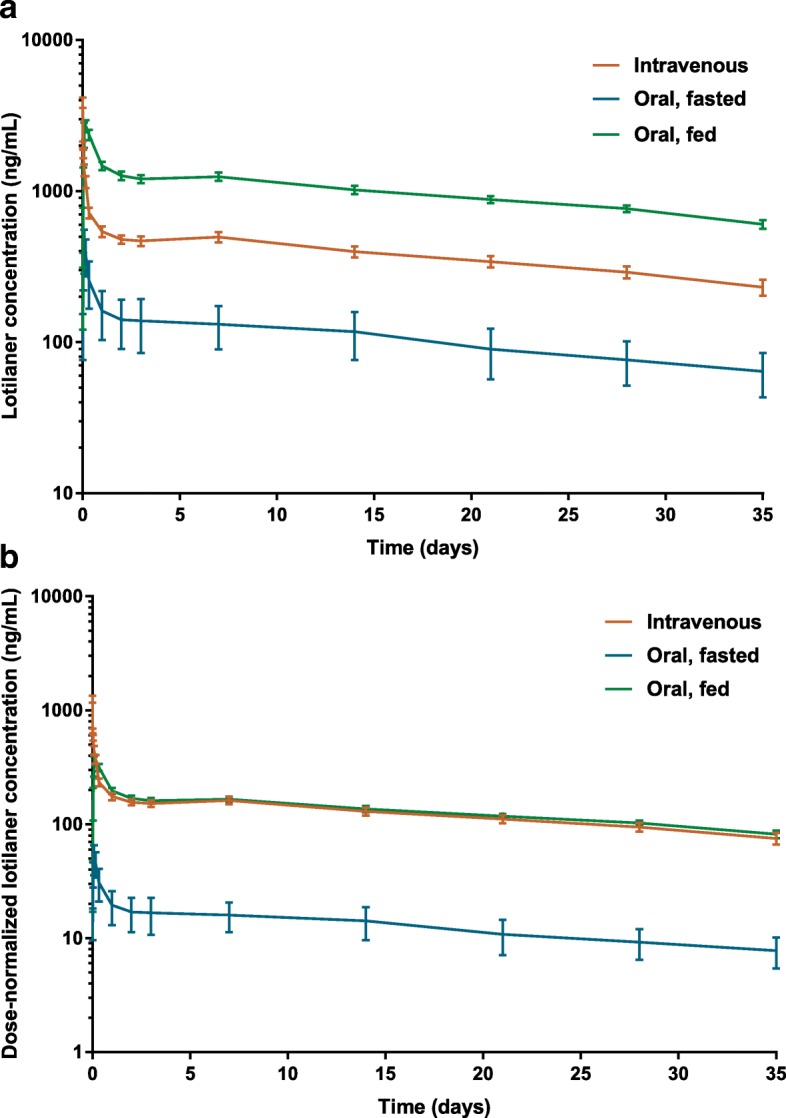
**a** Mean blood concentrations (± standard error) of lotilaner following intravenous or oral administration to fed or fasted cats, not dose-normalized. **b** Dose-normalized (to 1 mg/kg) mean blood concentrations (± standard error) of lotilaner following intravenous or oral administration to fed or fasted cats

The pharmacokinetic parameters of lotilaner are shown in Table 1 and drug concentrations *versus* time profiles after intravenous and oral administration under fed or fasted conditions are displayed in Fig. 1. The actual dose in the intravenous group ranged from 3.00 to 3.23 mg/kg (mean ± SD: 3.07 ± 0.07 mg/kg), in the oral-fed group from 6.44 to 8.92 mg/kg (mean ± SD: 7.52 ± 0.83 mg/kg), and in the oral-fasted group from 7.16 to 8.73 mg/kg (mean ± SD: 8.00 ± 0.70 mg/kg). All pharmacokinetic parameters presented are based on geometric means (considered as most appropriate, assuming that these parameters follow a log-normal distribution), except for Tmax which can only take discrete values and is therefore based on the median. A larger sample size was chosen for the oral-fed group, which represents the recommended route and prandial state for the commercial product, in order to obtain higher statistical power for this study group.

**Table 1:** Mean ± standard deviation pharmacokinetic parameters of lotilaner in adult cats after either single administration at a target dose of 6 mg/kg orally to fasted cats orally to fed cats, or at a target dose of 3.0 mg/kg intravenously. All values (mean and standard deviation) are based on geometric summary statistics (corresponding to summary statistics of log-transformed values and then back-transformed), except for T_max_ which is based on the median

Parameter	Intravenous, 3 mg/kg (*n* = 8)	Oral, fed 6 mg/kg (*n* = 11) ^b^	Oral, fasted 6 mg/kg (*n* = 6)
Actual doses (mg/kg)	3.07 ± 0.07	7.52 ± 0.83	8.00 ± 0.70
T_max_ (hours)	na	4 (range: 1–24)	2 (range: 1–4)
C_max_ (ng/ml)	na	3004 ± 709	348 ± 249
AUC_last_ (day*ng/ml)	13,238 ± 2839	33,907 ± 6611	2901 ± 2207
AUC_inf_ (day*ng/ml)	23,776 ± 9131	68,418 ± 23,220	4876 ± 3610
C_max_ (dose normalized) (ng/ml)^a^	na	403.4 ± 107.0	43.6 ± 28.6
AUC_last_ (dose normalized) (day*ng/ml)^a^	4315 ± 862	4554 ± 762	364 ± 255
AUC_inf_ (dose normalized) (day*ng/ml)^a^	7750 ± 2876	9188 ± 3632	612 ± 420
T_1/2z_ (day)	28.6 ± 14.3	33.6 ± 22.0	27.1 ± 7.7
MRT (day)	41.6 ± 19.5	48.9 ± 30.7	38.6 ± 9.9
CL (l/kg/day)	0.13 ± 0.05	na	na
V_z_ (l/kg)	5.34 ± 1.49	na	na
V_ss_ (l/kg)	5.37 ± 1.37	na	na
Bioavailability (F%)	na	106	8.4

^a^Dose-normalized to 1 mg/kg

^a^One cat had an adverse event unrelated to the treatment (leg fracture) and was withdrawn from the study

After intravenous administration at 3 mg/kg, a visual inspection of the profiles showed that lotilaner blood concentrations, as in dogs, decreased bi-exponentially with a rapid distribution phase and a long elimination phase (Fig. 1). The T_1/2z_ of lotilaner was 28.7 days and MRT was 41.6 days. Total blood clearance was 0.13 l/kg/day and the volumes of distribution V_z_ and V_ss_ were 5.34 and 5.37 l/kg, respectively. Mean dose-normalized AUC_last_ was 4315 day*ng/ml (Table 1).

After oral administration at 6 mg/kg, a visual inspection of the profiles showed that lotilaner blood concentrations decreased bi-exponentially after T_max_, with a rapid distribution phase within the first day of administration and a long elimination phase (Fig. 1). After oral administration in fed conditions, detectable blood levels were identified in all treated cats except one within 1 h indicating rapid dissolution and absorption of the tablet and maximum concentrations were observed at 4 h (T_max_) with mean dose-normalized C_max_ of 403 ng/ml. Concentrations declined with quantifiable blood concentrations for more than 5 weeks, with dose-normalized concentration 35 days after dosing at 82 ng/ml. The T_1/2z_ was 33.6 days and MRT was 48.9 days. Mean dose-normalized AUC_last_ (= AUC_0–35 d_) was 4554 day*ng/ml (Table 1). After oral administration in fasted conditions, significantly lower blood concentrations of lotilaner were observed with a mean dose-normalized C_max_ of 43.6 ng/ml and a T_max_ at 2 h. Mean dose-normalized AUC_last_ (= AUC_0-35 d_) was 364 day*ng/ml (Table 1). The mean T_1/2z_ after oral administration was in the same range as the one determined after intravenous administration, indicating that the terminal phase represented the true elimination phase. Relatively high variability between individuals was observed for T_1/2z_, especially when considering information from other studies. T_1/2z_ may vary between 15 and 40 days, and in a few cases even outside this range, but in all laboratory and field studies performed, this always remained without any impact on efficacy and safety.

For comparisons from the oral-fed and oral-fasted groups, differences in mean values for C_max_, AUC_last_, AUC_inf_ were significant and the difference in bioavailability (Table 1) between the oral-fed (106%) and oral-fasted groups (8.4%) was also significant (see Table 2 for detailed test statistics and exact *P*-values). The differences in T_1/2z_ and MRT between fed and fasted states were not statistically significant (Table 2). Moreover, the variability of the lotilaner pharmacokinetic parameters was moderate in the intravenous and oral-fed groups and much higher in the oral-fasted group. This high variability in the oral-fasted group was explained by the low bioavailability since this is known as a major source of variability [21]. In addition, in early pilot studies using non-commercial formulations, the reduction of the food ration to one-third of the daily ration did not impact the bioavailability and high bioavailability was achieved with both dry and wet food (unpublished data). Moreover, it was determined in a dog study that the exact time of feeding with respect to dosing (fed 30 min prior, fed at dosing, fed 30 min post-dosing) did not have a significant impact on bioavailability [14]. Lotilaner is acting as a systemic ectoparasiticide and parasites need to start feeding on the host to become exposed to the drug. Therefore, efficacy is directly correlated with blood concentrations and high bioavailability combined with low between-animal variability is critical to ensure consistent and sustained efficacy. In light of the significant difference between oral bioavailability in the fed compared to the fasted state, the product may not achieve an acceptable level of efficacy if administered to cats in the fasted state. The feeding effect on the bioavailability of lotilaner has previously been described in dogs [14] and has also been reported for other members of the isoxazoline family [22]. However, the need to administer lotilaner with food should not lead to practical inconvenience for the cat owner, since a small amount of food was found sufficient and the nature (wet *vs* dry food) turned out to be not critical. For example, the tablet can be administered hidden in a small amount of wet food before the usual daily feed ration is given to the cat. Hence a good understanding of pharmacokinetics has helped to predict how the drug should be administered in order to avoid cases of insufficient efficacy due to lower bioavailability.

**Table 2:** Comparisons between fed and fasted groups following oral administration of lotilaner at 6 mg/kg

Parameter	Ratio (fasted to fed)	90% CI	*t*-value	*P*-value^a^
T_1/2z_	0.806	0.503–1.291	*t*_(22)_ = -0.79	0.4397
MRT	0.789	0.504–1.234	*t*_(22)_ = -0.91	0.3730
C_max_ (dose normalized)	0.108	0.073–0.159	*t*_(15)_ = -10.05	< 0.0001
AUC_last_ (dose normalized)	0.080	0.058–0.110	*t*_(22)_ = -13.44	< 0.0001
AUC_inf_ (dose normalized)	0.067	0.044–0.100	*t*_(22)_ = -11.33	< 0.0001
Bioavailability	0.080	0.058–0.110	*t*_(22)_ = -13.44	< 0.0001

^a^*P*-values < 0.0001 denote significance

In order to interpret the clearance, the overall body extraction ratio was computed *via* the body clearance (0.13 l/kg/day) divided by the cardiac output (approximately 210 l/kg/day for a 3 kg cat) [23]. Hence, the total blood clearance corresponded to an overall extraction ratio of 0.06% and is therefore considered as very low. In addition, lotilaner had high volumes of distribution (5 l/kg), as expected for a lipophilic drug. The large volume of distribution together with the low clearance of lotilaner results in a long T_1/2z_ in the cat [24, 25]. This long T_1/2z_ and prolonged MRT account for the persistent systemic availability of lotilaner and maintains effective systemic concentrations for the entire duration of the inter-dosing interval of one month. Yet, the T_1/2z_, relative to the recommended dosing interval of one month, is not too long so that drug accumulation during repeated monthly administration remains moderate (accumulation ratio of approximately 2) [18].

Physiologically, the large volume of distribution can be explained by the tendency of the lipophilic substance to form a depot in fatty tissue. This has been experimentally verified in an absorption, distribution, metabolism and elimination (ADME) study with ^14^C radiolabelled lotilaner (unpublished data). This study also revealed that lotilaner is excreted mostly *via* the faeces with little metabolism. In blood and tissues, no or negligible presence of any metabolites was found.

Overall the presented data show some similarities of lotilaner pharmacokinetics in dogs and in cats. The group mean values of some key parameters in cats *versus* dogs compare as follows: blood clearance 0.13 *vs* 0.18 l/kg/day, volumes of distribution V_z_ 5.34 *vs* 6.35 l/kg and V_ss_ 5.37 *vs* 6.45 l/kg and T_1/2z_ 28.6 *vs* 24.6 days. A significant food effect was also present in both species and more pronounced in cats. Bioavailability was significantly higher under fed condition and much lower under fasted conditions: food enhanced bioavailability to > 80% in dogs and about 100% in cats whereas bioavailability was below 25% and 10% in fasted state for dogs and cats, respectively. Therefore, Credelio™ should be administered with food.

## Conclusions

Following a single intravenous administration, lotilaner had a long T_1/2z_ (28.7 days) resulting from a very low clearance (0.13 l/kg/day) and high volumes of distribution (5 l/kg). Following a single oral administration to fed cats, lotilaner blood concentrations peaked within four hours, had a long T_1/2z_ of 33.6 days, and had significantly greater bioavailability than when administered to fasted cats. Food (at least one third of the daily ration) enhanced the bioavailability to close to 100% and therefore it is recommended to administer tablets at or around the time of feeding. The observed long half-life of lotilaner and high bioavailability translated into blood concentrations remaining high enough to offer protection against fleas and *Ixodes ricinus* ticks for one month.

## Additional file

Additional file 1: French translation of the Abstract. (PDF 13 kb)

## Abbreviations

ADME: absorption, distribution, metabolism and elimination
ANOVA: analysis of variance
AUC_inf_: area under the blood concentration-time curve from zero to infinity
AUC_last_: area under the blood concentration-time curve from zero to the last time point for which blood concentration is above the limit of quantitation
CI: confidence interval
C_max_: maximum (peak) blood drug concentration
CL: total body clearance of drug from the blood
EMA: European Medicine Agency
F: female
FDA: Food and Drug Administration
GABACls: γ-aminobutyric acid (GABA)-gated chloride channels
HPLC: high-performance liquid chromatography
LC-MS/MS: liquid chromatography tandem mass spectrometry
LLOQ: lower limit of quantification
M: male
na: not applicable
SD: standard deviation
T_max_: time to reach maximum (peak) blood concentration following drug administration
T_1/2z_: elimination half-life associated with the terminal slope of a semi-logarithmic concentration time curve
V_z_: volume of distribution during the terminal phase
V_ss_: volume of distribution at steady-state
