# Supplementary material for: Pharmacokinetics of lotilaner following a single oral or intravenous administration in cats
Source: Parasit Vectors. 2018 Jul 13;11:412. doi: 10.1186/s13071-018-2966-6 (PMC6044034; doi:10.1186/s13071-018-2966-6)
Supplement: Supplementary file 1 — French translation of the Abstract. (PDF 13 kb) [file 13071_2018_2966_MOESM1_ESM.pdf]

Pharmacocinétique du lotilaner après administration d'une dose unique par voie orale ou intraveineuse chez le chat

Céline E Toutain[1*], Wolfgang Seewald[1] et Martin Jung[1]

[1]Elanco Animal Health, Mattenstrasse 24a, CH-4058, Bâle, Suisse

*Correspondance : celine.toutain@elanco.com

Résumé

Contexte : Credelio™ (lotilaner) est un antiparasitaire externe de la classe des isoxazolines, administré par voie orale et développé contre les infestations par les tiques et les puces chez le chat. Il se présente sous forme de comprimés à croquer aromatisés, à base d'énantiomère (*S*) pur. Les propriétés pharmacocinétiques du lotilaner ont été étudiées après administration par voie orale ou intraveineuse chez des chats à jeun ou non. Vingt-six chats adultes ont été inclus dans une étude pharmacocinétique évaluant l'administration intraveineuse ou orale du lotilaner. Suite à l'administration orale du produit à raison de 6 mg/kg, ou intraveineuse à 3 mg/kg, chez des animaux à jeun ou non, des échantillons de sang ont été prélevés jusqu'à 35 jours après le traitement. Les concentrations sanguines en lotilaner ont été dosées selon une méthode validée de chromatographie en phase liquide couplée à la spectrométrie de masse. Les paramètres pharmacocinétiques ont été calculés par une analyse non compartimentale. La stabilité *in vivo* de l'énantiomère du lotilaner a également été évaluée dans le cadre d'une étude bio-analytique distincte.

Résultats : Suite à l'administration par voie orale chez des chats nourris, le lotilaner a été rapidement absorbé, le pic de concentration sanguines ayant été atteint dans les 4 heures. La demi-vie a été de 33,6 jours. La prise d'un repas a favorisé l'absorption et permis d'atteindre une biodisponibilité orale proche de 100 %, tout en réduisant la variabilité interindividuelle. Suite à son administration intraveineuse, le lotilaner a présenté une clairance faible de 0,13 l/kg/jour, d'importants volumes de distribution V_z et V_{ss}**,** respectivement de 5,34 et 5,37 l/kg, et une demi-vie de 28,7 jours. De plus, aucune racémisation du lotilaner n'a été observée *in vivo*.

Conclusions : les propriétés pharmacocinétiques du lotilaner, administré par voie orale sous forme de comprimé à croquer aromatisé (Credelio™), ont fait l'objet d'une étude approfondie. Avec un T_{max} de 4 heures et une demi-vie de 33,6 jours chez les animaux nourris, le lotilaner exerce un effet létal rapide sur les puces et les tiques, son efficacité étant continue et prolongée pendant au moins un mois chez le chat.

Mots clés : lotilaner, isoxazoline, pharmacocinétique, chat, voie orale, voie intraveineuse, effet de l'alimentation, à jeun, non à jeun
